# GRIPHIN: grids of pharmacophore interaction fields for affinity prediction

**DOI:** 10.1186/s13321-026-01203-8

**Published:** 2026-05-07

**Authors:** Daniel Rose, Thomas Seidel, Thierry Langer

**Affiliations:** 1 Department of Pharmaceutical Sciences, Division of Pharmaceutical Chemistry, Faculty of Life Sciences, University of Vienna, Josef-Holaubek-Platz 2, 1090 Vienna, Austria; 2 Christian Doppler Laboratory for Molecular Informatics in the Biosciences, Department of Pharmaceutical Sciences, University of Vienna, Josef-Holaubek-Platz 2, 1090 Vienna, Austria; 3 Vienna Doctoral School of Pharmaceutical, Nutritional and Sport Sciences, University of Vienna, Josef-Holaubek-Platz 2, 1090 Vienna, Austria

## Abstract

Pharmacophores are widely used to describe protein-ligand interactions. In this work, we propose a hybrid framework for binding affinity prediction that combines pharmacophoric maps of the protein binding site with a graph-based representation of the ligand. Our method achieves performance comparable to state-of-the-art models while offering interpretability through attribution methods, thereby demonstrating the potential of pharmacophoric representations in deep learning.

**Scientific contribution**

We investigate whether a purely pharmacophoric representation of the protein pocket is sufficient to train a deep learning model for affinity prediction. For this purpose, we devise a hybrid model architecture from simple building blocks for affinity prediction. To enhance interpretability, we apply integrated gradients to attribute predictions to individual pharmacophoric features. Source code and model weights are available at https://github.com/molinfo-vienna/GRIPHIN.

## Introduction

In recent years, a substantial body of deep learning methodologies has been proposed for bioactivity prediction [1]. Early work predominantly employed voxel-based architectures [2–7], motivated by the success of convolutional neural networks (CNNs) in image classification [8]. These approaches typically encode the binding site as a discretized, ligand-centered three-dimensional grid, with differences among methods arising primarily from input preprocessing choices that determine the model’s inductive biases. Due to the strong performance of graph neural networks (GNNs) in molecular property prediction [9], research focus shifted toward graph-based methods for affinity prediction, where proposed model architectures vary mainly in their graph construction strategies and graph convolutional update rules [10–12].

While most research remains heavily focused on developing novel architectures, there is not much work on benchmark datasets, and most models are trained and evaluated on the PDBbind [13]. Since new methods are primarily judged by their ability to outperform existing baselines, we observe a trend toward increasingly complex architectures that incorporate auxiliary sources of information, such as scoring-function terms [14], language-model embeddings [15], and surface descriptors [16]. However, over-reliance on benchmark performance for architectural design decisions is problematic for the following reasons. Benchmarks are susceptible to adaptive overfitting, as new designs are inherently informed by prior results [17]. Furthermore, the approximately 20,000 data points in the PDBbind 2020 version are relatively few for deep learning, making the risk of overfitting significant. While data leakage in the standard core set split [18, 19] has led to the proposal of alternative splits [15, 20], model evaluation remains constrained by the use of a single dataset. Given that generating new experimental data in cheminformatics is resource-intensive, we must acknowledge the limitations of our current hold-out sets. Rather than asking how to build more advanced architectures to marginalize gains on a stagnant benchmark, we propose a different question: How can we simplify our model architecture while still achieving competitive results?

We address this question by exploring *Grids of Pharmacophore Interaction Fields* (GRAIL) [21] as model input, a simple and interpretable voxel-based pharmacophoric representation of binding pockets. Pharmacophores are abstract representations of the supramolecular interactions between proteins and ligands derived from steric and electronic molecular features of the ligand and proximal residues of the binding site [22]. For practitioners, the GRAIL representation provides insights into potential binding modes, while for machine learning applications, it introduces an inductive bias that focuses models on relevant interaction types without requiring explicit learning of amino acid residue properties. While recent work by Fellinger et al. [23] demonstrated the utility of GRAIL-based fingerprints for binding affinity prediction, the full potential of GRAIL maps in combination with modern deep learning remains unexplored. Although existing studies have utilized pharmacophoric representations for binding site prediction [24] or contrastive learning of protein descriptors [25], there is currently no deep learning model for binding affinity prediction based exclusively on a pharmacophoric protein representation.

**Fig. 1:**
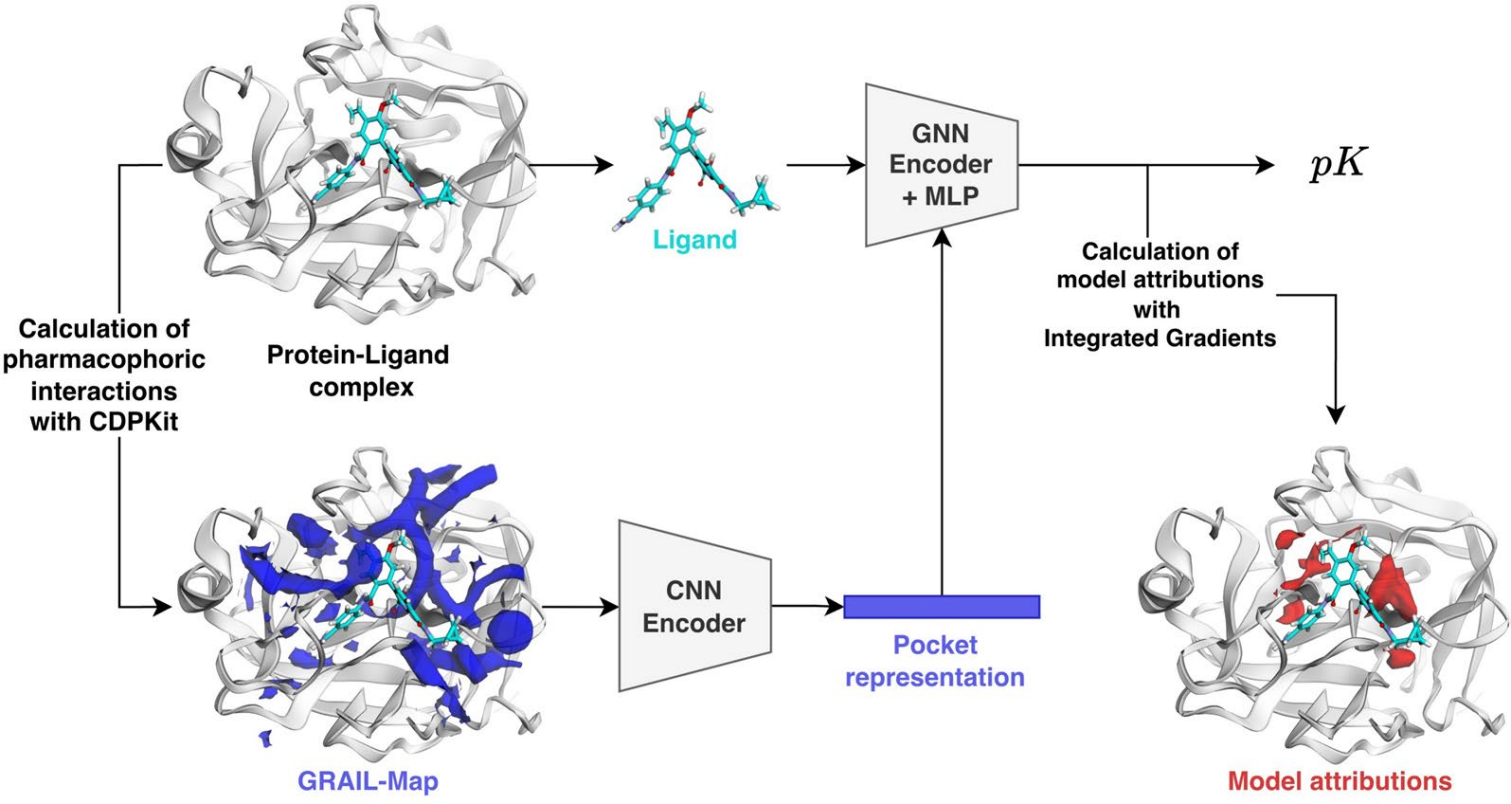
GRIPHIN takes a protein-ligand complex as input, the protein is provided as a PDB file and the ligand as an SD file. The GRAIL map is computed for the protein using the standard implementation provided by the CDPKit [26] and processed by a 3D CNN encoder to generate a pocket representation, serving as context vector during ligand encoding. The resulting interaction representation is used to predict the binding affinity *pK*. Integrated gradients [27] attribution is computed using the Captum Python package [28] to identify regions of the GRAIL map that are most relevant to the model’s predictions. The attribution highlights key areas influencing the regression model. The presented example was randomly selected from the validation set (PDB code: 6BFP)

We present GRIPHIN (*Gri*ds of *Ph*armacophore *In*teraction fields for affinity prediction), a hybrid framework that integrates voxel-based pharmacophoric GRAIL representations of the binding site with graph-based ligand representations. An overview of this methodology is displayed in Fig. 1. By using GRAIL maps, we introduce an intuitive inductive bias aligned with the pharmacophore concept, thereby simplifying the input representation while maintaining high interpretability. While the primary focus of this paper is the design and evaluation of the architecture, we find that coupling attribution methods with human-interpretable GRAIL maps offers a promising avenue for identifying interaction hotspots and guiding lead optimization.

## Methods

### Overview

**Fig. 2:**
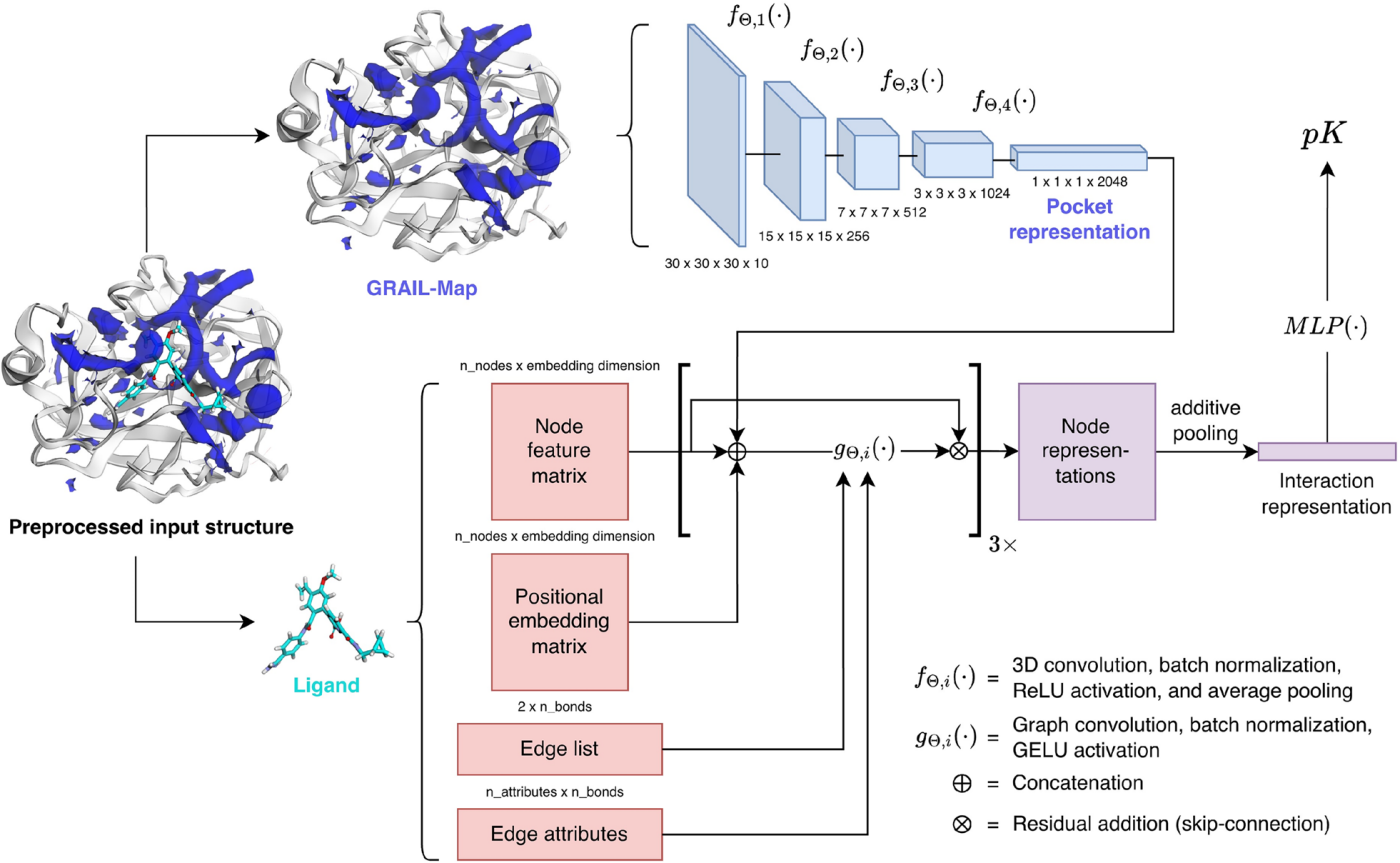
GRIPHIN transforms the GRAIL map into a learned representation, using a 3D-CNN encoder consisting of four layers with increasing filter sizes, batch normalization, ReLU activation, and average pooling. The resulting pocket representation is concatenated as a context vector to each row of the ligand’s node feature matrix, along with the positional encoding of the Cartesian coordinates of the corresponding atoms. The node representations are updated through three iterations of graph convolution layers, each followed by batch normalization, GELU activation, and a residual connection to the features from the previous layer. The updated node representations are then aggregated into a graph-level interaction representation using additive pooling. Finally, the *pK* value is predicted using a multi-layer perceptron (MLP)

The GRIPHIN model processes the GRAIL map of the protein pocket using a 3D-CNN to generate a pocket representation. This pocket representation serves as a context vector for the ligand encoder, which is a GNN that takes the molecular graph of the ligand as input. The initial node representations of the ligand graph are constructed by concatenating atom type embeddings, a positional encoding of the Cartesian coordinates of the node positions, and the context vector. Through repeated graph convolution operations, the model generates an interaction representation, which is passed to the final prediction head to produce the regression output. An overview of our proposed model architecture is given in Fig. 2.

### Data

The PDBbind dataset [13] is a curated collection of annotated protein-ligand complexes. For this work, we use the PDBbind v.2020 version, encompassing a total of 19,443 data points. Each data point consists of a PDB file representing the protein and an SD file representing the ligand. We use the provided files without additional preprocessing. The affinity values in the dataset are provided as $K_D$, $K_i$, or $IC_{50}$ values. Following the protocol described in Jiménez et al. [2], we do not differentiate between these types of measurements. Instead, we convert all values to $pK = -\log _{10}K$ and use these as the target values throughout this work.

### Pharmacophoric interaction fields

Inspired by Goodford’s GRID method [29–31], GRAIL uses pharmacophoric feature points to probe the complementarity of ligand-side features within a rasterized grid of the binding site. Each grid point is evaluated for its potential to host specific ligand features, such as hydrogen-bond donors, acceptors, hydrophobic areas, or aromatic systems. Interactions are modeled through generalized bell functions and calculated from distances and angles relative to the protein residues. Individual residue contributions are aggregated at each grid point by either selecting the maximum contribution to model the presence of interaction types or by summation for capturing interaction hotspots. The inclusion of angular dependencies is important for modeling directed pharmacophoric features such as hydrogen bonds and aromatic $\pi$-stacking and depicts a unique feature of GRAIL maps over other methods, which usually use radial distances only for modeling pharmacophoric interactions. Finally, atom density grids are computed to account for steric clashes, reducing interaction scores at grid points near or within the van der Waals radius of receptor atoms.

### Input processing

For each protein-ligand complex, the GRAIL map interaction channels are computed from the corresponding PDB file using the functionality provided by CDPKit [26]. The CDPKit standard implementation centers the grid map on the ligand and extends the bounding box by 7 Å in each direction to include relevant binding pocket residues in the GRAIL computation. To process the voxelized GRAIL maps with the 3D-CNN encoder, we fix the input dimensions to 15 Å in each direction and set the voxel edge length to 0.5 Å, resulting in a 30 $\times$ 30 $\times$ 30 voxel grid. If the calculated grid is smaller than 15 Å in any dimension, the input tensor is padded with zeros.

### Pocket representation

The pocket encoder adopts the standard structure of CNNs. The input to the encoder is the voxelized GRAIL map tensor $\textbf{P}$ with dimensions $\mathbb {R}^{k \times k \times k \times C}$, where *k* is the voxel grid size and *C* is the number of channels. In our implementation, we set $k = 30$, and the standard CDPKit implementation provides $C = 10$ distinct channels. The GRAIL map channels are designed to scale between [0, 1], except for the lipophilic interaction channel. This exception arises because the lipophilic interaction score regards the hydrophobic weight of the features involved in the interaction. The hydrophobic weight is computed by summing the lipophilicity contributions of the atoms covered by the feature which may result in values exceeding 1. For a more detailed explanation of the composition of GRAIL interaction channels, readers are referred to the original publication by Schütz et al. [21]. To address this, we introduce a learnable scaling factor for each channel, which is applied before the convolutional layers to allow flexible rescaling. The convolutional layers follow a standard architecture, where the input channels are convolved with learnable kernels of size 3, stride length 1, and padding. Each convolution is followed by batch normalization, non-linear ReLU activation, and pooling. For our use case, we found that average pooling outperforms max pooling. A total of four convolutional modules $f_{\Theta , i}: \mathbb {R}^{k \times k \times k \times s_i} \rightarrow \mathbb {R}^{(k/2) \times (k/2) \times (k/2) \times s_{i+1}}$ produces the final pocket representation vector $\textbf{c} \in \mathbb {R}^s$, where *s* is the final vector dimension. This pocket representation serves as the context vector for the subsequent interaction encoding step.

### Ligand representation

The ligand is represented as an undirected attributed graph $G = (V, E, \lambda )$, where *V* is the set of nodes, $E \subseteq V \times V$ is the set of undirected edges, and $\lambda : V \cup E \rightarrow \mathcal {L}$ assigns attributes from the label set $\mathcal {L}$ to the nodes and edges. We are following the convention of PyTorch Geometric [32] and represent *G* as the tuple $\textbf{m} = (\textbf{x}, \textbf{r}, \textbf{a}, \textbf{e})$, where $\textbf{x} \in \mathbb {R}^{n \times q}$ is the node feature matrix with $n = |V|$ nodes and *q* feature dimensions (one-hot encoded atom types), $\textbf{r} \in \mathbb {R}^{n \times 3}$ are the spatial positions of the nodes within the GRAIL grid, $\textbf{a} \in \mathbb {R}^{2 \times m}$ is the edge list with $m = |E|$ edges (each column specifying the indices of the two connected nodes), and $\textbf{e} \in \mathbb {R}^{m \times u}$ is the edge feature matrix with *u* feature dimensions (one-hot encodings of bond order, aromaticity, and ring membership). The node attributes $\lambda (v)$ are encoded as rows of $\textbf{x}$, and edge attributes $\lambda (e)$ are encoded as rows of $\textbf{e}$.

### Positional encoding

Incorporating positional information is essential for contextualizing the atomic nodes relative to the global pocket descriptor. While Cartesian coordinates can be directly used as node features, prior research indicates that deep learning models often achieve improved performance when positional encodings are employed. In our model, the spatial positions of nodes are encoded using Fourier features [33], defined as:

$$\begin{aligned} \mathbf {\gamma }(\textbf{r})&= [\ldots , \sin (\nu _i\textbf{r}), \cos (\nu _i\textbf{r}), \ldots ] \quad \text {for} \; i = 0, \ldots , d-1, \\ \nu _i&= \exp \left( -\log (1000) \cdot \frac{2i}{d}\right) , \end{aligned}$$

where $\mathbf {\gamma }(\textbf{r}) \in \mathbb {R}^{n \times 3 \cdot d}$ represents the positional encodings of dimension *d* for the three Cartesian coordinates, and $\nu _i$ denotes the log-spaced frequency at index *i*. This approach to positional encoding has been previously utilized in various contexts, such as the 1D sequential positional encodings in transformer models by Vaswani et al. [34] and the reconstruction of 3D protein structures by Zhong et al. [35]. Additionally, we explored the use of radial basis functions as an alternative positional encoding method, achieving comparable results.

### Interaction encoding

The interaction encoding is structured as follows: The ligand’s OHE node features $\textbf{x}$ are passed through an initial embedding layer, producing node embeddings $\textbf{h}_0 \in \mathbb {R}^{n \times l}$, where *n* is the number of nodes and *l* is the dimension of the hidden features. The node positions $\textbf{r} \in \mathbb {R}^{n \times 3}$ are encoded using the described positional encoding, resulting in Fourier features $\mathbf {\gamma }(\textbf{r}) \in \mathbb {R}^{n \times 3 \cdot d}$, where $3\cdot d$ is the dimension of the positional encodings. Additionally, the context vector $\textbf{c} \in \mathbb {R}^s$ from the pocket encoder is broadcast to match the node dimensionality *n*, yielding context representations $\textbf{c}' \in \mathbb {R}^{n \times s}$. At each graph convolution iteration, the positional embeddings $\mathbf {\gamma }(\textbf{r})$ and context representations $\textbf{c}'$ are concatenated with the node embeddings $\textbf{h}_i$ from the *i*th layer. The resulting features are processed by a graph convolution layer using the graph attention mechanism described in Velickovic et al. [36] and Brody et al. [37]. The output of the convolution is passed through batch normalization and a GELU activation function, and then added residually to the input node representations $\textbf{h}_i$ to produce the updated node embeddings $\textbf{h}_{i+1}$. This process is repeated for three graph convolution modules $g_{\Theta , i}: \mathbb {R}^{l+s+3 \cdot d} \rightarrow \mathbb {R}^l$. The final node representations $\textbf{h}_3$ are aggregated into a graph-level interaction representation $\textbf{z} \in \mathbb {R}^l$ by summing along the node dimension. Finally, a projection head, implemented as a two-layer multi-layer perceptron (MLP), maps the interaction representation $\textbf{z}$ to the predicted *pK* value for the regression task.

### Model training

The model is trained using the mean squared error (MSE) loss and the AdamW optimizer [38], with an initial learning rate of $1 \times 10^{-3}$ and a weight decay of $1 \times 10^{-6}$. We found that augmentation of the training data by randomly reflecting the GRAIL maps and the corresponding ligand coordinates along the coordinate axes led to better convergence. The CNN model consists of four convolutional layers, with an initial embedding dimension of $s_0 = 256$ and a final output dimension of $s = 2048$. The hidden dimension of the GNN node representations is set to $l = 1024$, and the projection head is implemented as a two-layer MLP with a hidden dimension of $l_{\text {MLP}} = 4096$. The model was trained for 600 epochs on a randomly generated training and validation split, with validation performance on this split used for hyperparameter selection. An effective batch size of 256 was used during training. A training run on an NVIDIA RTX 4090 GPU required approximately 12 h. We trained the final model configuration with five different random seeds and report the mean and standard deviation of the respective evaluation metrics. Details on model training and hyperparameters ranges explored during optimization are available in the Supporting Information S.2.

### Attribution method

Given a neural network $F: \mathbb {R}^n \rightarrow \mathbb {R}$ and an input $\textbf{x} \in \mathbb {R}^n$, an attribution method produces a contribution vector $A_F(\textbf{x}, \textbf{x}') = \textbf{a} \in \mathbb {R}^n$, which quantifies the importance of each input feature $x_i$ relative to a baseline $x_i'$ with respect to the model output. The baseline represents the absence of a signal, typically provided as an empty input vector. We employ the Integrated Gradients method [27], a computationally efficient and straightforward post-hoc approach, as implemented in the Captum library [28]. This method interpolates linearly between the baseline and the input, accumulating gradients along the interpolation path:

1 $$\begin{aligned} \textrm{IntegratedGrads}_i := (x_i - x_i') \int _{\alpha =0}^{1} \frac{\partial F(\textbf{x}' + \alpha (\textbf{x} - \textbf{x}'))}{\partial x_i} \, d\alpha , \end{aligned}$$

where $\alpha \in [0, 1]$ is the interpolation parameter, and $\frac{\partial F(\textbf{x})}{\partial x_i}$ is the gradient of the model output $F(\textbf{x})$ with respect to the input feature $x_i$. Intuitively, the gradient measures the sensitivity of the model output to changes in the input feature. The cumulative sum of gradients along the interpolation path, scaled by the difference between the input and baseline, quantifies the feature’s contribution to the prediction. We use a GRAIL map with all zero values as the baseline, representing the absence of pharmacophoric interactions. Since gradients on our model’s output depend on both the GRAIL map and the ligand, the attribution values of the GRAIL map are conditioned on the ligand input, yielding a subset that highlights regions the model associates with its prediction. We use these attributions qualitatively to visualize potential areas of relevance. For visualization, attribution values are normalized by dividing by the highest absolute contribution value.

## Results and discussion

### Overview

We evaluate our model on the PDBbind core set [18], PDBbind CleanSplit [15], and the Leak-Proof PDBbind [20] split, together with three additional test sets proposed by Li et al. [20], and identify critical design choices of our model via ablations. We then employ the integrated gradients method to highlight important regions in the voxel box, given the model’s predictions. This information is used to create a visualization of the pharmacophoric interaction. We conclude our discussion with limitations and potential future directions for our work.

### Data splits

The PDBbind core set is a well-established data split of the PDBbind and was introduced in the *Comparative Assessment of Scoring Functions* (CASF-2016) benchmark [18, 19] for evaluating scoring functions in structure-based drug design. It contains 290 high-resolution protein-ligand complexes, spanning 58 proteins and five ligands. However, recent studies showed that results derived from this benchmark should be interpreted with caution. Francoeur et al. [3] conducted clustered cross-validation on PDBbind and observed a performance drop, which they attributed to the overlap between the chemical and target spaces of the core set used for evaluation and of the refined and general sets used for training. They concluded that evaluations based on the core set might be overly optimistic. To create a more challenging data split for the PDBbind dataset, Li et al. [20] proposed a splitting strategy based on ligand and protein similarity. This new split, called Leak-Proof PDBbind (LP-PDBbind), consists of 11,513/2,422/4,860 data points in the training/validation/test sets and was used to retrain four popular models. They additionally proposed three independent test sets that are not derived from the PDBbind dataset. The BDB2020+ test set consists of 115 data points from BindingDB [39], deposited after 2020, with experimental binding affinity data retrieved from the RCSB Protein Data Bank [40]. Two target-specific test sets were also introduced: the Mpro test set, containing 40 ligands binding to the SARS-CoV-2 main protease, and the EGFR test set, comprising 23 ligands bound to the epidermal growth factor receptor. A complementary approach to LP-PDBbind is PDBbind CleanSplit by Graber et al. [15]. Instead of defining a new test set, it retains the CASF-2016 core set and removes training entries likely to cause data leakage, reducing the number of complexes in the combined general and refined set of PDBbind from 19,153 to 16,908 complexes for training and validation.

### Performance evaluation

Table 1 summarizes the performance of various binding affinity prediction models on the CASF-2016 core set, evaluated using the Pearson correlation coefficient (PCC), mean squared error (MSE), mean absolute error (MAE), and root mean squared error (RMSE). A short description of the presented baselines can be found in the Supporting Information S.1. While some architectures achieve a PCC exceeding 0.83, the majority of affinity prediction models in current literature report values approximately at or below 0.82. Our model performs in line with these metrics, demonstrating comparable results to recent studies. We thereby demonstrate that good performance is achievable with a minimal pocket representation that is conceptually aligned with the pharmacophore concept. A further advantage of our hybrid model architecture over purely voxel-based methods is that we are not constrained by ligand size.

T-Alpha exemplifies the shift toward complex multimodal architectures, integrating dMaSIF-like surface descriptors, E(3)-equivariant GNNs on structural graphs, transformer encodings of protein sequences and SMILES, and physicochemical features. It reports a comparatively high $\text {PCC}=0.87$, but at the cost of substantial architectural complexity. GraphscoreDTA likewise attains a slightly higher PCC by combining Vina terms with protein, ligand, and interaction encoders. While these gains are commendable, they may partly reflect overfitting on the comparable small PDBbind dataset. Moreover, maximal test-set performance is not always preferable in practice when simplicity and interpretability are desired. According to Occam’s razor, when multiple methods achieve similar performance, the conceptually simpler one is preferable.

**Table 1:** Comparison of model performance

Model	PCC $\uparrow$	MSE $\downarrow$	MAE $\downarrow$	RMSE $\downarrow$
GraphBAR$^*$ [10]	0.73	–	1.24	1.54
pafnucy$^*$ [6]	0.78	–	1.13	1.42
PIGNet$^\dagger$ [11]	0.79	–	–	–
sfcnn$^\dagger$ [7]	0.79	–	1.03	1.32
Def2018 General Ensemble$^*$ [3]	0.80	–	–	1.37
midlevel fusion$^*$ [41]	0.81	–	1.02	1.31
OnionNet$^*$ [42]	0.82	$\mathbf {1.63}$	0.98	–
$\Delta _{Vina}RF_{20}$ $^*$ [18]	0.82	–	–	–
PLAIG$^\ddagger$ [43]	0.82	1.69	1.01	–
KDeep$^*$ [2]	0.82	–	–	1.27
PLANET$^\ddagger$ [12]	0.82	–	0.97	1.25
GraphscoreDTA$^\dagger$ [14]	0.83	–	0.98	1.25
T-Alpha$^\ddagger$ [16]	$\mathbf {0.87}$	–	$\mathbf {0.88}$	$\mathbf {1.11}$
GRIPHIN (Ours)$^\ddagger$	$0.80 \pm 0.01$	$1.68 \pm 0.09$	$0.95 \pm 0.03$	$1.30 \pm 0.04$

We report test set performance on the CASF-2016 core set in terms of PCC, MSE, MAE, and RMSE. Performance values of other work is stated as reported in the respective publications. PDBbind version used for training: v.2016 ($^*$), v.2019 ($^\dagger$), v.2020 ($^\ddagger$). For the GRIPHIN model, we report the mean and standard deviation over five models with different random initialization. The best entries are highlighted in bold

Table 2 summarizes results on the LP-PDBbind test sets, and correlation plots between the true and predicted values are provided in Supporting Information S.4. The split was designed to mitigate data leakage, and as expected, GRIPHIN’s performance decreases on LP-PDBbind relative to CASF-2016. While still T-Alpha [16] achieves the highest performance, its advantage is less pronounced than on the CASF-16 core set. The Mpro test set represents complexes similar to the training distribution, since PDBbind includes related SARS-CoV-1 protease proteins, while the EGFR test set represents complexes that differ from the training distribution [20]. Interestingly, our model performs better on EGFR than on Mpro. Although two external sets are insufficient for definitive conclusions about generalization, the overall comparison to published baselines indicates reasonable performance compared to the other affinity predictors.

**Table 2:** Comparison of model performance across datasets

Model	LP-PDBbind		BDB2020+		Mpro		EGFR	
	PCC $\uparrow$	RMSE $\downarrow$	PCC $\uparrow$	RMSE $\downarrow$	PCC $\uparrow$	RMSE $\downarrow$	PCC $\uparrow$	RMSE $\downarrow$
AutoDock Vina [44]	–	1.88	0.29	1.54	0.66	0.86	0.38	1.17
IGN [45]	–	1.58	0.54	1.01	0.61	1.06	0.65	0.70
RF-Score [46]	–	1.54	0.51	1.18	0.52	1.20	0.52	0.71
DeepDTA [47]	–	1.68	0.26	1.26	0.64	0.65	0.44	0.77
T-Alpha [16]	$\mathbf {0.55}$	$\mathbf {1.50}$	$\mathbf {0.68}$	$\mathbf {0.97}$	$\mathbf {0.72}$	$\mathbf {0.65}$	**0.70**	**0.69**
GRIPHIN (Ours)	$0.50 \pm 0.01$	$1.60 \pm 0.03$	$0.38 \pm 0.06$	$1.34 \pm 0.15$	$0.43 \pm 0.07$	$1.09 \pm 0.10$	$0.58 \pm 0.15$	$0.93\pm 0.12$

For each dataset, we report test set performance in terms of PCC and RMSE. Performance values of other work are stated as reported in the respective publications. For the GRIPHIN model, we report the mean and standard deviation over five models with different random initialization﻿. The best entries are highlighted in bold

To complete our evaluation, we retrained and tested GRIPHIN on the PDBbind CleanSplit [15]. Graber et al. [15] retrained Pafnucy and GenScore on CleanSplit alongside their GEMS model and included a nearest-neighbor baseline that averages the affinities of the five most similar training complexes. Results are summarized in Table 3. While GRIPHIN’s performance on the new split is slightly lower than on the original split, it remains within a competitive range and performs comparably to Pafnucy. Although GEMS achieves the highest scores, it benefited from specific optimization for this split. The CleanSplit authors note that GEMS required iterative refinements and the integration of language embeddings to reach these metrics. Conversely, GRIPHIN maintained reasonable performance using its original hyperparameters, achieving these results without further optimization.

Finally, to evaluate the importance of pharmacophoric features, we trained a GRIPHIN variant using only ligand information, excluding the GRAIL map. Removing the protein context vector caused performance to drop to the nearest-neighbor baseline. This decline confirms that the context provided by the pharmacophoric maps is essential for accurate predictions. In summary, we evaluated GRIPHIN under three test-set strategies and observed consistently reasonable performance. The model’s primary strength is its simple, interpretable representation of pharmacophoric interaction patterns. We contend that there is no one-size-fits-all solution for affinity prediction and that a modest trade-off in accuracy for greater interpretability should be advantageous in many applications.

**Table 3:** Comparison of model performance on the PDBbind CleanSplit [15]

Model	PCC $\uparrow$	RMSE $\downarrow$
Search top 5 complexes [15]	0.65	1.65
Pafnucy (retrained on PDBbind v.2020) [6]	0.75	1.48
GenScore [48]	0.78	1.36
GEMS [15]	$\mathbf {0.80}$	$\mathbf {1.31}$
GRIPHIN (Ours)	$0.76 \pm 0.01$	$1.42 \pm 0.03$
GRIPHIN (Ligand information only)	$0.65 \pm 0.02$	$1.67 \pm 0.03$

Performance values of other work are stated as reported by Graber et al. [15]. For the GRIPHIN model, we report the mean and standard deviation over five models with different random initialization﻿. The best entries are highlighted in bold

### Ablation studies

To identify the critical components of our model, we conduct ablation studies by evaluating performance degradation on the validation set of the PDBbind v.2020 core split. Detailed results of the ablations are presented in Table S2 in the Supporting Information S.3. The context vector, which holds information about the protein pocket, is the most important component of the model. Omitting it is equivalent to training a model on ligand information only and leads to the largest drop in validation performance from 1.48 to 1.94 MSE. Using context during graph convolution is also crucial. If the context vector is concatenated to the graph representation before the MLP, instead of to the node representations before convolution, a performance drop to 1.70 MSE is observed. The MLP used to map the graph-level representation to the affinity value is another key component. A simple linear layer is insufficient for this task, highlighting the importance of non-linear activation after graph convolution. The depth and width of the MLP are less critical, and we found that a depth of two with a hidden dimension of 4096 provides the best results. Additive pooling outperforms mean pooling when aggregating node representations into a graph-level representation, which is consistent with findings in other works on molecular property prediction. The choice of graph convolution layer proved to be less critical overall. Among the tested options, the GAT layer delivered the best performance. While the GINE layer also performed well, GAT was selected due to the anticipated advantages of its attention mechanism in integrating the context vector. For the CNN architecture, increasing the hidden dimensions and the number of layers both improves performance. We settled on 256 hidden dimensions for the first convolutional filter and four CNN layers, which provides a good balance between performance and the number of model parameters. Interestingly, incorporating positional embeddings of the atom coordinates results in only a small improvement. This suggests that the molecular graph and the pocket descriptor already capture most of the information required for the prediction task.

**Fig. 3:**
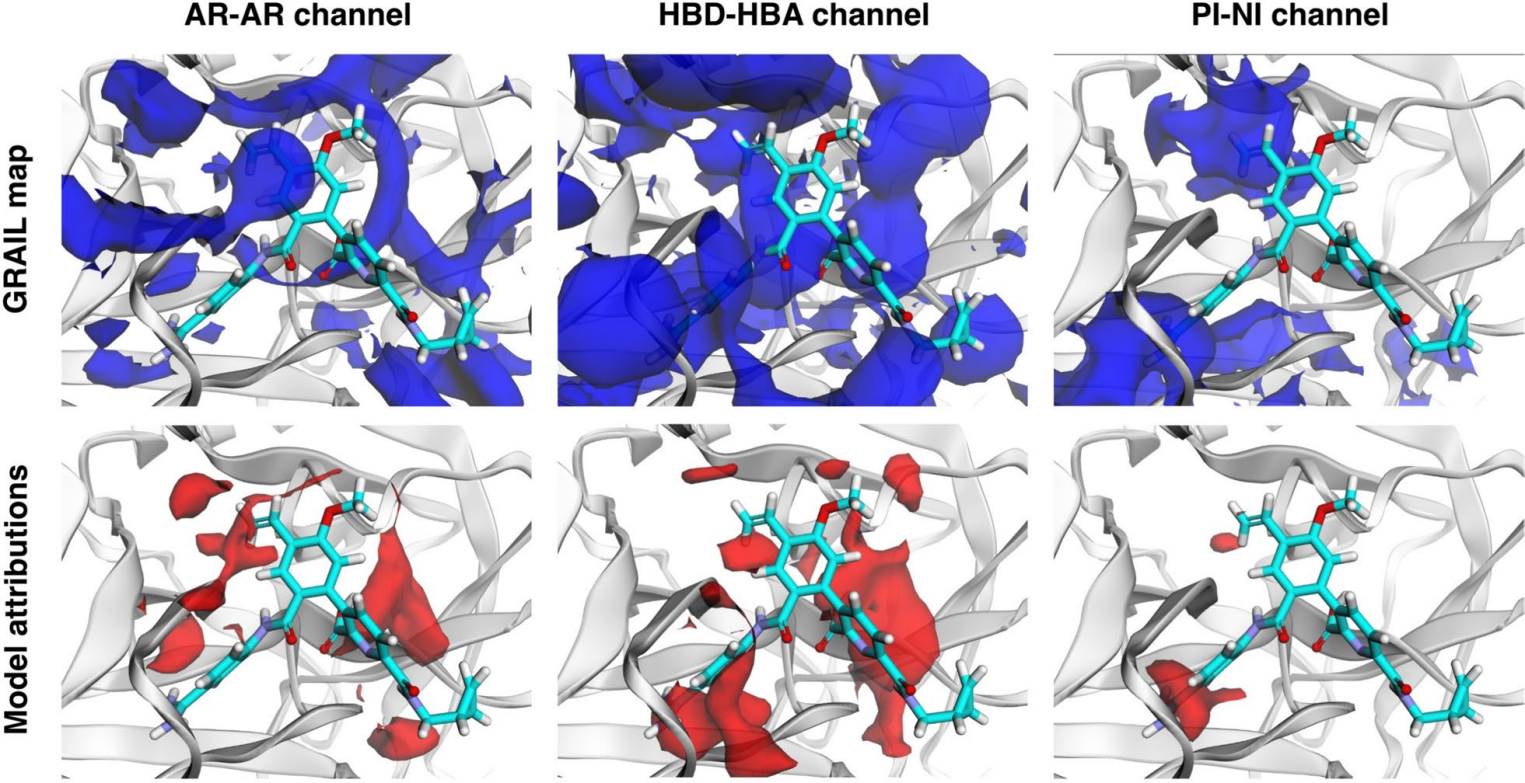
Visualization of three example channels (top, blue) and the corresponding attribution values (bottom, red) for the target 6BFP, randomly selected from the validation set. Pharmacophoric features are categorized as hydrophobic (H), aromatic (AR), positive ionizable (PI), negative ionizable (NI), hydrogen bond donor (HBD), hydrogen bond acceptor (HBA), halogen bond donor (XBD), halogen bond acceptor (XBA). Channel notation follows the convention of [ligand-feature]-[binding-site-feature], for example, the HBD-HBA channel indicates that a ligand hydrogen bond donor is energetically favorable given the hydrogen bond acceptor moieties present in the binding site. Visualizations of all ten GRAIL channels can be found in the Supporting Information S.5

### Model interpretability

Schütz et al. [21] demonstrated that GRAIL maps effectively highlight key interaction areas within a protein pocket, providing valuable insights for practitioners. However, because GRAIL computes interactions across the entire voxel grid, the resulting visualization can become overly complex and difficult to interpret. Additionally, it represents only the pocket without accounting for the ligand, limiting its contextual relevance. By incorporating an attribution method, the model produces ligand-conditioned highlights on the GRAIL map, assigning attribution values to identify regions relevant to its prediction. High, positive values indicate input voxels that increase the prediction relative to an empty baseline voxel box. Figure 3 illustrates this effect by displaying several GRAIL channels for a randomly selected validation target, further examples are included in the Supporting Information S.5. These visualizations highlight potential ligand–site complementarity, with ligand-conditioned attributions rescaling the map to emphasize regions the model associates with its prediction. While this does not imply the ligand must possess the indicated pharmacophoric features, it suggests such features could enhance the predicted *pK* value. This capability holds promise for lead optimization, where attributions could guide experts in modifying an initial ligand pose. Overall, we view this as a promising step toward practical interpretability, while recognizing that additional case studies are needed to establish actionable value for practitioners.

### Limitations

Our model takes as input the GRAIL representation of the binding pocket, a feature-engineered and human-interpretable representation. While this format enhances visualization and interpretability, it limits model training to predefined channels. Although inductive bias enables models to learn from fewer data points, it constrains the information that can be extracted from the input. Additionally, machine learning models struggle to extrapolate beyond the domain of their training data. The PDBbind database provides valuable training data, but its size remains comparably small for fully leveraging the capacity of deep learning models. Expanding the dataset would likely improve the performance and generalizability of GRIPHIN. Moreover, while attribution methods offer useful insights for interpreting model predictions, their reliability in specific scenarios is difficult to assess and should be approached with caution. Despite these limitations, we believe that our model, in its current form, represents a valuable addition to the toolkit of affinity prediction methods in drug discovery and serves as a robust foundation for future advancements.

### Outlook

Future work will focus on leveraging model attributions to develop a principled workflow for lead optimization. Furthermore, our model could be deployed as a scoring function in docking pipelines. Due to its close alignment with the pharmacophore concept, we anticipate that such application could complement 3D pharmacophore-based virtual screening. Beyond screening, conditioning 3D molecular generation on the GRAIL representation of the protein pocket is a promising avenue. The associated interpretability would enable practitioners to directly evaluate the plausibility of proposed 3D ligand structures. Another direction could be to incorporate receptor flexibility via molecular dynamics (MD), which may enhance affinity prediction. Schütz et al. [21] combined GRAIL with protein dynamics by averaging interaction-type-specific maps over MD trajectories, thereby revealing stable and transient regions of the binding pocket. However, applying this at scale would require generating an MD trajectory for each protein–ligand complex in the PDBbind, incurring substantial computational cost. Methodologically, the learning problem would shift toward a multi-instance framework, for which reported gains in chemistry applications are mixed and typically modest [49]. A possible advantage of this extension would be the ability to identify the most probable active conformation along an MD trajectory. Overall, while integrating flexibility is scientifically compelling, its practical utility must be weighed against the significant computational overhead.

## Conclusion

In this study, we explore the use of pharmacophoric fields for affinity prediction. We present a hybrid framework for protein-ligand binding affinity prediction that combines voxel-based GRAIL maps of protein binding pockets with graph-based ligand representations. The proposed GRIPHIN model achieves good predictive performance, as demonstrated on the CASF-2016, LP-PDBbind, and PDBbind CleanSplit benchmarks, while offering interpretability through its use of intuitive pharmacophoric interaction fields. We show that GRAIL maps can be conditioned on the ligand using attribution methods, providing a more informative visualization of potential binding modes. We hope that our study motivates further studies towards model simplification, against the trend of increasingly complex model architectures, and to explore model designs with practical advantages beyond the maximization of predictive accuracy.

## Additional file

Supplementary file 1 (pdf 4370 KB)

## Data Availability

The code implementation of this project is open-source and available under https://github.com/molinfo-vienna/GRIPHIN. The software is platform-independent, written in Python, and distributed under the MIT license. The repository contains code for model training, evaluation, and notebooks for visualization purposes. It further contains scripts for data processing, and the data splits used in this study, including the CASF-2016 core set. The pretrained models are available via figshare https://figshare.com/articles/journal_contribution/GRIPHIN_Grids_of_Pharmacophore_Interaction_Fields_for_Affinity_Prediction_-_Trained_model_and_preprocessed_data/30272203, together with the preprocessed BDB2020+, mpro, and EGFR test datasets of the LP-PDBbind study, which were downloaded from the respective repository https://github.com/THGLab/LP-PDBbind. The PDBbind v.2020 dataset, which was used for model training, cannot be provided by us for download due to licensing restrictions. However, it is available free of charge and can be downloaded after registration from the official PDBbind website https://www.pdbbind-plus.org.cn/. The implementation of our data processing pipeline is available in our code repository.

## Declarations

### Supporting information

Supporting information is available free of charge and includes a related works section on voxel-based methods, graph-based methods, model interpretability, and data split details (Section S.1), detailed information on model training, the full list of hyperparameters of the final model, and implementation details (Section S.2, Table S1), details on the ablation study (Section S.3, Table S2), correlation plots of the model predictions on the CASF-2016 core set, the LP-PDBbind test set, and additional test sets as provided by the LP-PDBbind study (Section S.4, Figs. S1, S2), and three example complexes to visualize the ten different GRAIL channels and the corresponding model attributions (Section S.5, Figs. S3–S5).

## References

[1] Debnath K, Rana P, Ghosh P (2025) A survey on deep learning for drug-target binding prediction: models, benchmarks, evaluation, and case studies. Brief Bioinform 26:bbaf491 40977267 10.1093/bib/bbaf491 PMC12451107

[2] Jiménez J, Skalic M, Martinez-Rosell G, De Fabritiis G (2018) K deep: protein-ligand absolute binding affinity prediction via 3D-convolutional neural networks. J Chem Inf Model 58:287–296 29309725 10.1021/acs.jcim.7b00650

[3] Francoeur PG, Masuda T, Sunseri J, Jia A, Iovanisci RB, Snyder I, Koes DR (2020) Three-dimensional convolutional neural networks and a cross-docked data set for structure-based drug design. J Chem Inf Model 60:4200–4215 32865404 10.1021/acs.jcim.0c00411 PMC8902699

[4] McNutt AT, Francoeur P, Aggarwal R, Masuda T, Meli R, Ragoza M, Sunseri J, Koes DR (2021) GNINA 1.0: molecular docking with deep learning. J Cheminform 13:43 34108002 10.1186/s13321-021-00522-2 PMC8191141

[5] Ragoza M, Hochuli J, Idrobo E, Sunseri J, Koes DR (2017) Protein-ligand scoring with convolutional neural networks. J Chem Inf Model 57:942–957 28368587 10.1021/acs.jcim.6b00740 PMC5479431

[6] Stepniewska-Dziubinska MM, Zielenkiewicz P, Siedlecki P (2018) Development and evaluation of a deep learning model for protein-ligand binding affinity prediction. Bioinformatics 34:3666–3674 29757353 10.1093/bioinformatics/bty374 PMC6198856

[7] Wang Y, Wei Z, Xi L (2022) Sfcnn: a novel scoring function based on 3D convolutional neural network for accurate and stable protein-ligand affinity prediction. BMC Bioinform 23:222 10.1186/s12859-022-04762-3 PMC9178885 35676617

[8] Krizhevsky A, Sutskever I, Hinton GE (2012) ImageNet classification with deep convolutional neural networks. In: Proceedings of the 25th conference on neural information processing systems (NIPS)

[9] Wieder O, Kohlbacher S, Kuenemann M, Garon A, Ducrot P, Seidel T, Langer T (2020) A compact review of molecular property prediction with graph neural networks. Drug Discov Today Technol 37:1–12 34895648 10.1016/j.ddtec.2020.11.009

[10] Son J, Kim D (2021) Development of a graph convolutional neural network model for efficient prediction of protein-ligand binding affinities. PLoS ONE 16:e0249404 33831016 10.1371/journal.pone.0249404 PMC8031450

[11] Moon S, Zhung W, Yang S, Lim J, Kim WY (2022) PIGNet: a physics-informed deep learning model toward generalized drug-target interaction predictions. Chem Sci 13:3661–3673 35432900 10.1039/d1sc06946b PMC8966633

[12] Zhang X, Gao H, Wang H, Chen Z, Zhang Z, Chen X, Li Y, Qi Y, Wang R (2023) Planet: a multi-objective graph neural network model for protein-ligand binding affinity prediction. J Chem Inf Model 64:2205–2220 37319418 10.1021/acs.jcim.3c00253

[13] Liu Z, Su M, Han L, Liu J, Yang Q, Li Y, Wang R (2017) Forging the basis for developing protein-ligand interaction scoring functions. Acc Chem Res 50:302–309 28182403 10.1021/acs.accounts.6b00491

[14] Wang K, Zhou R, Tang J, Li M (2023) GraphscoreDTA: optimized graph neural network for protein-ligand binding affinity prediction. Bioinformatics 39:btad340 37225408 10.1093/bioinformatics/btad340 PMC10243863

[15] Graber D, Stockinger P, Meyer F, Mishra S, Horn C, Buller R (2025) Resolving data bias improves generalization in binding affinity prediction. Nat Mach Intell 7:1713–1725 41143208 10.1038/s42256-025-01124-5 PMC12552120

[16] Kyro GW, Smaldone AM, Shee Y, Xu C, Batista VS (2025) T-ALPHA: a hierarchical transformer-based deep neural network for protein-ligand binding affinity prediction with uncertainty-aware self-learning for protein-specific alignment. J Chem Inf Model 65:2395–2415 39965912 10.1021/acs.jcim.4c02332

[17] Dwork C, Feldman V, Hardt M, Pitassi T, Reingold O, Roth A (2015) Generalization in adaptive data analysis and holdout reuse. In: Proceedings of the 28th conference on neural information processing systems (NIPS)

[18] Su M, Yang Q, Du Y, Feng G, Liu Z, Li Y, Wang R (2018) Comparative assessment of scoring functions: the CASF-2016 update. J Chem Inf Model 59:895–913 30481020 10.1021/acs.jcim.8b00545

[19] Li Y, Su M, Liu Z, Li J, Liu J, Han L, Wang R (2018) Assessing protein-ligand interaction scoring functions with the CASF-2013 benchmark. Nat Protoc 13:666–680 29517771 10.1038/nprot.2017.114

[20] Li J, Guan X, Zhang O, Sun K, Wang Y, Bagni D, Head-Gordon T (2026) Leak proof PDBBind: a reorganized data set of protein-ligand complexes for more generalizable binding affinity prediction. J Phys Chem B 130:730–740 (**PMID: 41486605**) 41486605 10.1021/acs.jpcb.5c08598 PMC13430955

[21] Schütz DA, Seidel T, Garon A, Martini R, Körbel M, Ecker GF, Langer T (2018) GRAIL: grids of pharmacophore interaction fields. J Chem Theory Comput 14:4958–4970 30075621 10.1021/acs.jctc.8b00495

[22] Wermuth C-G, Ganellin CR, Lindberg P, Mitscher LA (1998) Glossary of terms used in medicinal chemistry (IUPAC Recommendations 1998). Pure Appl Chem 70:1129–1143

[23] Fellinger C, Seidel T, Merget B, Schleifer K-J, Langer T (2025) GRADE and X-GRADE: unveiling novel protein-ligand interaction fingerprints based on GRAIL scores. J Chem Inf Model 65:2456–2475 39980202 10.1021/acs.jcim.4c01902 PMC11898076

[24] Jiménez J, Doerr S, Martínez-Rosell G, Rose AS, De Fabritiis G (2017) DeepSite: protein-binding site predictor using 3D-convolutional neural networks. Bioinformatics 33:3036–3042 28575181 10.1093/bioinformatics/btx350

[25] Simonovsky M, Meyers J (2020) DeeplyTough: learning structural comparison of protein binding sites. J Chem Inf Model 60:2356–2366 32023053 10.1021/acs.jcim.9b00554

[26] Seidel T (2025) Chemical data processing toolkit source code repository. https://github.com/molinfo-vienna/CDPKit. Accessed 18 Aug 2025

[27] Sundararajan M, Taly A, Yan Q (2017) Axiomatic attribution for deep networks. In: Proceedings of the 34th international conference on machine learning (ICML), pp 3319–3328

[28] Kokhlikyan N, Miglani V, Martin M, Wang E, Alsallakh B, Reynolds J, Melnikov A, Kliushkina N, Araya C, Yan S et al (2020) Captum: a unified and generic model interpretability library for pytorch. arXiv:2009.07896

[29] Goodford PJ (1985) A computational procedure for determining energetically favorable binding sites on biologically important macromolecules. J Med Chem 28:849–857 3892003 10.1021/jm00145a002

[30] Boobbyer DN, Goodford PJ, McWhinnie PM, Wade RC (1989) New hydrogen-bond potentials for use in determining energetically favorable binding sites on molecules of known structure. J Med Chem 32:1083–1094 2709375 10.1021/jm00125a025

[31] Wade RC, Clark KJ, Goodford PJ (1993) Further development of hydrogen bond functions for use in determining energetically favorable binding sites on molecules of known structure. 1. Ligand probe groups with the ability to form two hydrogen bonds. J Med Chem 36:140–147 8421280 10.1021/jm00053a018

[32] Fey M, Lenssen JE (2019) Fast graph representation learning with PyTorch geometric. arXiv:1903.02428

[33] Tancik M, Srinivasan P, Mildenhall B, Fridovich-Keil S, Raghavan N, Singhal U, Ramamoorthi R, Barron J, Ng R (2020) Fourier features let networks learn high frequency functions in low dimensional domains. In: Proceedings of the 34th conference on neural information processing systems (NIPS), pp 7537–7547

[34] Vaswani A, Shazeer N, Parmar N, Uszkoreit J, Jones L, Gomez AN, Kaiser Ł, Polosukhin I (2017) Attention is all you need. In: Proceedings of the 31st conference on neural information processing systems (NIPS), pp 6000–6010

[35] Zhong ED, Bepler T, Davis JH , Berger B (2020) Reconstructing continuous distributions of 3D protein structure from cryo-EM images. In: Proceedings of the 8th international conference on learning representations (ICLR)

[36] Velickovic P, Cucurull G, Casanova A, Romero A, Liò P, Bengio Y (2018) Graph attention networks. In: Proceedings of the 6th international conference on learning representations (ICLR)

[37] Brody S , Alon U, Yahav E (2022) How attentive are graph attention networks? In: Proceedings of the 10th international conference on learning representations (ICLR)

[38] Loshchilov I, Hutter F (2019) Decoupled weight decay regularization. In: Proceedings of the 7th international conference on learning representations (ICLR)

[39] Liu T, Lin Y, Wen X, Jorissen RN, Gilson MK (2007) BindingDB: a web-accessible database of experimentally determined protein-ligand binding affinities. Nucleic Acids Res 35:D198–D201 17145705 10.1093/nar/gkl999 PMC1751547

[40] Burley SK, Bhikadiya C, Bi C, Bittrich S, Chao H, Chen L, Craig PA, Crichlow GV, Dalenberg K, Duarte JM et al (2023) RCSB protein data bank (RCSB.org): delivery of experimentally-determined PDB structures alongside one million computed structure models of proteins from artificial intelligence/machine learning. Nucleic Acids Res 51:D488–D508 36420884 10.1093/nar/gkac1077 PMC9825554

[41] Jones D, Kim H, Zhang X, Zemla A, Stevenson G, Bennett WD, Kirshner D, Wong SE, Lightstone FC, Allen JE (2021) Improved protein-ligand binding affinity prediction with structure-based deep fusion inference. J Chem Inf Model 61:1583–1592 33754707 10.1021/acs.jcim.0c01306

[42] Zheng L, Fan J, Mu Y (2019) OnionNet: a multiple-layer intermolecular-contact-based convolutional neural network for protein-ligand binding affinity prediction. ACS Omega 4:15956–15965 31592466 10.1021/acsomega.9b01997 PMC6776976

[43] Samudrala MV, Dandibhotla S, Kaneriya A, Dakshanamurthy S (2025) PLAIG: protein-ligand binding affinity prediction using a novel interaction-based graph neural network framework. ACS Bio & Med Chem Au 5:447–463 10.1021/acsbiomedchemau.5c00053 PMC12183606 40556781

[44] Trott O, Olson AJ (2010) AutoDock Vina: improving the speed and accuracy of docking with a new scoring function, efficient optimization, and multithreading. J Comput Chem 31:455–461 19499576 10.1002/jcc.21334 PMC3041641

[45] Jiang D, Hsieh C-Y, Wu Z, Kang Y, Wang J, Wang E, Liao B, Shen C, Xu L, Wu J et al (2021) InteractionGraphNet: a novel and efficient deep graph representation learning framework for accurate protein-ligand interaction predictions. J Med Chem 64:18209–18232 34878785 10.1021/acs.jmedchem.1c01830

[46] Ballester PJ, Mitchell JB (2010) A machine learning approach to predicting protein-ligand binding affinity with applications to molecular docking. Bioinformatics 26:1169–1175 20236947 10.1093/bioinformatics/btq112 PMC3524828

[47] Öztürk H, Özgür A, Ozkirimli E (2018) DeepDTA: deep drug–target binding affinity prediction. Bioinformatics 34:i821–i829 30423097 10.1093/bioinformatics/bty593 PMC6129291

[48] Shen C, Zhang X, Hsieh C-Y, Deng Y, Wang D, Xu L, Wu J, Li D, Kang Y, Hou T et al (2023) A generalized protein-ligand scoring framework with balanced scoring, docking, ranking and screening powers. Chem Sci 14:8129–8146 37538816 10.1039/d3sc02044d PMC10395315

[49] Zankov DV, Matveieva M, Nikonenko AV, Nugmanov RI, Baskin II, Varnek A, Polishchuk P, Madzhidov TI (2021) QSAR modeling based on conformation ensembles using a multi-instance learning approach. J Chem Inf Model 61:4913–4923 34554736 10.1021/acs.jcim.1c00692

