# Supplementary material for: GRIPHIN: grids of pharmacophore interaction fields for affinity prediction
Source: J Cheminform. 2026 May 7;18:97. doi: 10.1186/s13321-026-01203-8 (PMC13371655; doi:10.1186/s13321-026-01203-8)
Supplement: Supplementary file 1 — (pdf 4370 KB) [file 13321_2026_1203_MOESM1_ESM.pdf]

# – Supporting Information –

GRIPHIN: Grids of Pharmacophore Interaction Fields for Affinity Prediction

Daniel Rose,[†,‡,¶] Thomas Seidel,[*,†,‡] and Thierry Langer[†,‡]

†*Department of Pharmaceutical Sciences, Division of Pharmaceutical Chemistry, Faculty of Life Sciences, University of Vienna, Josef-Holaubek-Platz 2, 1090 Vienna, Austria*

‡*Christian Doppler Laboratory for Molecular Informatics in the Biosciences, Department of Pharmaceutical Sciences, University of Vienna, Josef-Holaubek-Platz 2, 1090 Vienna, Austria*

¶*Vienna Doctoral School of Pharmaceutical, Nutritional and Sport Sciences, University of Vienna, Josef-Holaubek-Platz 2, 1090 Vienna, Austria*

E-mail: thomas.seidel@univie.ac.at

S.1 Related works

Voxel-based methods Voxel-based approaches represent the binding site as a discretized 3D grid, typically centered on the ligand with a cubic box of approximately 20 Å side length and a resolution of 0.5–1.0 Å. The K_{Deep} model[1] constructs the voxel grid using van der Waals radii of ligand and protein atoms, assigning atom types to distinct interaction channels. Similarly, the Def2018 model,[2] part of the GNINA scoring function family,[3] builds a 3D grid of Gaussian-like atom densities with 14 ligand atom type and 14 receptor atom type channels, processed by a 3D CNN for pose ranking and evaluation. This model builds on earlier work by Ragoza et al.[4]. The pafnucy model[5] encodes atoms using 19 features, including one-hot-encoded (OHE) atom types, hybridization states, bond counts, pharmacophoric properties, partial charges, and a protein/ligand atom flag. The Sfcnn model[6] employs an even simpler featurization, summing OHE atom types of ligand and protein within each voxel, using 14 channels for each. Although not explored for affinity prediction, several studies have employed purely pharmacophoric protein representations in deep learning. Early work includes DeepSite,[7] which uses a convolutional neural network (CNN) with eight pharmacophoric channels to predict protein binding sites. The same input representation was later adopted in DeeplyTough[8] for contrastive learning of pocket descriptors. Additionally, DeepGRID[9] combined Goodford's GRID method with deep learning, using GRID-derived descriptors and a CNN to predict blood–brain barrier (BBB) permeation.

Graph-based methods These models typically represent proteins as residue-level or atom-level graphs, ligands as molecular graphs, and their interactions through interaction edges or interaction graphs. The GraphBAR model[10] constructs a graph from ligand atoms and protein atoms within 4 Å of the ligand, using node features such as atom types, hybridization, valence, and partial charge. Multiple adjacency matrices with varying distance cutoffs are employed to capture different interaction ranges. PIGNet[11] uses atom types and two

adjacency matrices, one for covalent bonds and another for intermolecular interactions, to parametrize physics-informed equations for binding affinity prediction. The PLANET model[12] leverages more advanced graph neural network (GNN) techniques, representing the binding pocket as a 3D graph of protein residues in combination with a modified version of the equivariant GNN (EGNN) by Satorras et al.[13]. The ligand is modeled as a 2D graph, and protein-ligand interactions are updated via cross-attention. Similarly, GraphscoreDTA[14] employs a protein-residue graph, a molecular graph, and a pocket-ligand distance-dependent interaction graph, which are processed by a GNN with attention blocks and gated recurrent units. The T-ALPHA model[15] integrates multimodal descriptors to enrich protein, ligand, and interaction representations. Protein features are derived from dMaSIF-inspired surface descriptors,[16] structural graphs processed by an E(3)-EGNN, and transformer-based sequence embeddings. Ligand features are encoded using physicochemical descriptors, structural graphs with an EGNN, and SMILES-based transformers. Interactions are modeled with an EGNN, and all modalities are fused via cross-attention.

Model interpretability Explainable artificial intelligence (XAI) aims to make the decision-making processes of machine learning models understandable to humans. Some models, like transformers with attention mechanisms, possess intrinsic interpretability, but most architectures do not. Post-hoc attribution methods address this by assigning importance scores to input features, neurons, or layers, quantifying their contributions to the model's output. Perturbation-based methods alter input features and measure the resulting output changes, whereas gradient-based methods leverage backpropagation to assess the sensitivity of the output to variations in the input. Several affinity prediction models have explored interpretability techniques. CNN-Score[4] uses a masking strategy to visualize important regions, Pafnucy[5] employs weight distributions of the first convolutional layer and a voxel-removal strategy, Sfcnn[6] applies Grad-CAM to highlight critical input regions, and GraphscoreDTA[14] uses attention maps to visualize the contribution of residues and ligand atoms.

Data split details The *Comparative Assessment of Scoring Functions* (CASF-2016) benchmark[17,18] is widely adopted for evaluating scoring functions in structure-based drug design. A key contribution of this study is the introduction of the PDBbind core set, a well-established data split of the PDBbind. It contains 290 high-resolution protein-ligand complexes, spanning 58 proteins and five ligands. CASF-2016 also defines four evaluation metrics: scoring power, ranking power, docking power, and screening power. Scoring power, the focus of this study, assesses a model's ability to predict binding affinities for known protein-ligand complexes by computing the Pearson correlation coefficient (PCC) between predicted binding scores and experimental binding constants. While assuming the availability of experimentally resolved complexes depicts an idealized scenario, such tests serve as a critical baseline for evaluating new model architectures. Scoring functions that fail to perform well in this controlled setting are unlikely to generalize effectively to practical applications involving docked poses. The top-performing model in the scoring power benchmark from the original study is the $\Delta_{Vina}RF_{20}$ scoring function.[19] This model is a random forest-based approach that combines 20 molecular descriptors with the AutoDock Vina score.[20]

To create a more challenging data split for the PDBbind dataset, Li et al.[21] proposed a splitting strategy based on ligand and protein similarity, where ligand similarity was quantified using the Dice similarity of Morgan fingerprints,[22] while protein similarity was defined as the percentage of matched residues after sequence alignment using the Needleman-Wunsch[23] algorithm. In their approach, the training set was restricted to data points with a maximum protein sequence similarity of 0.5 and a ligand similarity of 0.99 relative to the validation and test sets. Similarly, the validation set was constrained to data points with a protein similarity of no more than 0.9 and a ligand similarity of no more than 0.99 when compared to the test set. This new split, termed Leak-Proof PDBbind (LP-PDBbind), consists of 11,513/2,422/4,860 data points in the training/validation/test sets and was used to retrain four popular models. The PDBbind CleanSplit by Graber et al.[24] retains the CASF-2016 core set and removes

training entries likely to cause data leakage. CleanSplit clusters structures using protein similarity (TM-score), ligand similarity (Tanimoto), and binding-conformation similarity (pocket-aligned ligand RMSD), reducing the number of complexes in the combined general and refined set of PDBbind from 19,153 to 16,908 complexes for training and validation.

S.2 Model training

Hyper parameter optimization For model testing, we use the core set test split as proposed by the CASF-2016 publication, which is comprised of 290 data samples. The remaining data is split randomly with a fixed seed of 42 into training and validation data with a ratio of 90:10. We perform hyper parameter optimization by random selection of model parameters and pick the best performing model based on the MSE on the validation set. The hyper parameters of the final model are summarized in Table S1.

Table S1: Hyperparameters of the final model after optimization.

**Parameter**	**Value**
CNN number of channels in the first layer	256
CNN number of layers	4
GNN convolution operator	GATv2
GNN convolution operator output dimension	1024
GNN number of layers	3
MLP dimension hidden layers	4096
MLP number of hidden layers	2
batch size	256
dropout factor	0.3
exclusion of hydrogen atoms	false
learning rate	0.001
maxmimum number of epochs	600
node pooling	add
dimension of positional embedding	240
weight_decay	1.0e-06

Implementation details Our code is implemented using Python v.3.11.13, development was performed on a Rocky Linux v.9.4 operating system. For model implementation, we use Pytorch (v.2.7.1+cu128), Pytorch Geometric (v.2.6.1), and Pytorch Lightning (v.2.5.1). Chemical data processing and GRAIL map calculations were performed using CDPKit (v.1.2.3) functionality. Attribution methods are used as implemented in Captum (v.0.8.0).

S.3 Ablation studies

For ablations, we fixed the hyper parameters of our model and retrained it while changing only one parameter. Table S2 shows the influence of changing model parameters on the model performance on the validation set.

Table S2: Ablation study results for different model components. The reported values correspond to the MSE performance metric on the validation set (random split with seed 42).

**Component**	**Configuration**	**Performance**
CNN first layer	32, 64, 128, 256	1.74, 1.63, 1.53, 1.48
CNN layers	2, 3, 4	1.66, 1.63, 1.48
MLP dimension	4096, 2048, 1024, 128	1.48, 1.48, 1.48, 1.53
MLP layers	0, 1, 2, 3, 4	1.92, 1.53, 1.48, 1.50, 1.50
GNN hidden dim	256, 512, 1024	1.52, 1.50, 1.48
Pooling	Add, Mean	1.48, 1.50
Context	Before, After, None	1.48, 1.70, 1.94
Layer	GATv1, GATv2, GINE, TransformerConv	1.49, 1.48, 1.50, 1.50
Pos Embedding dim	0, 240	1.49, 1.48

S.4 Correlation plots

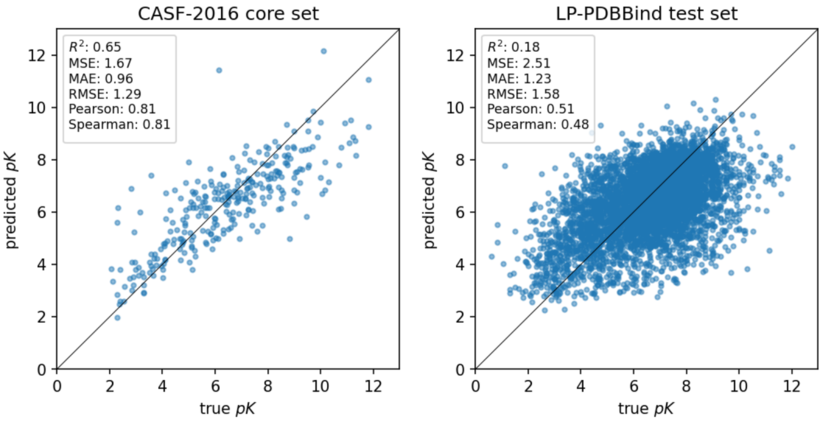

Figure S1: Test set performance of the GRIPHIN model (random seed 42) on the CASF-16 core set and the test set of the LP-PDBbind split. For testing the core set performance, the model was trained on a random training/validation split of the general set. For testing on the LP-PDBbind dataset, we used the training/validation split as provided by the authors.

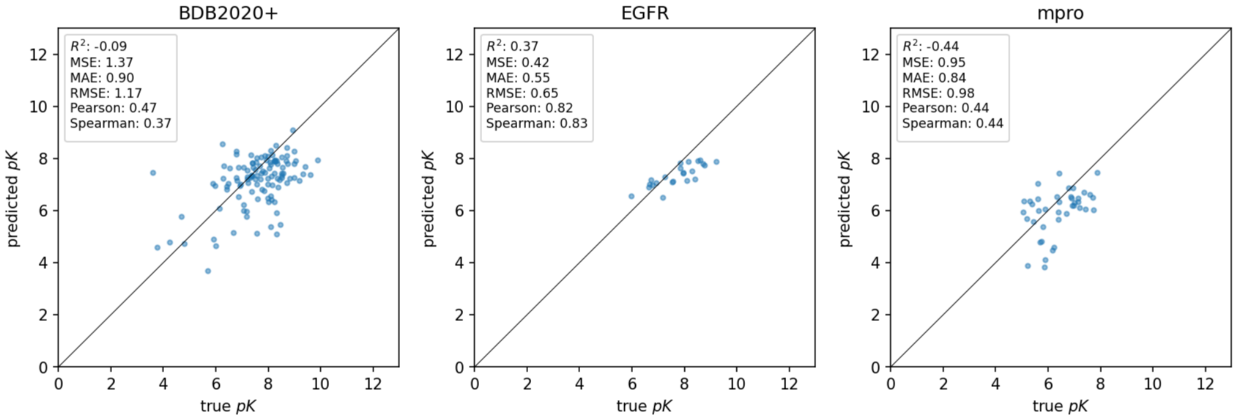

Figure S2: Test performance of the GRIPHIN model (random seed 42) on the three additional test sets provided by the LP-PDBbind publication.

S.5 GRAIL visualization

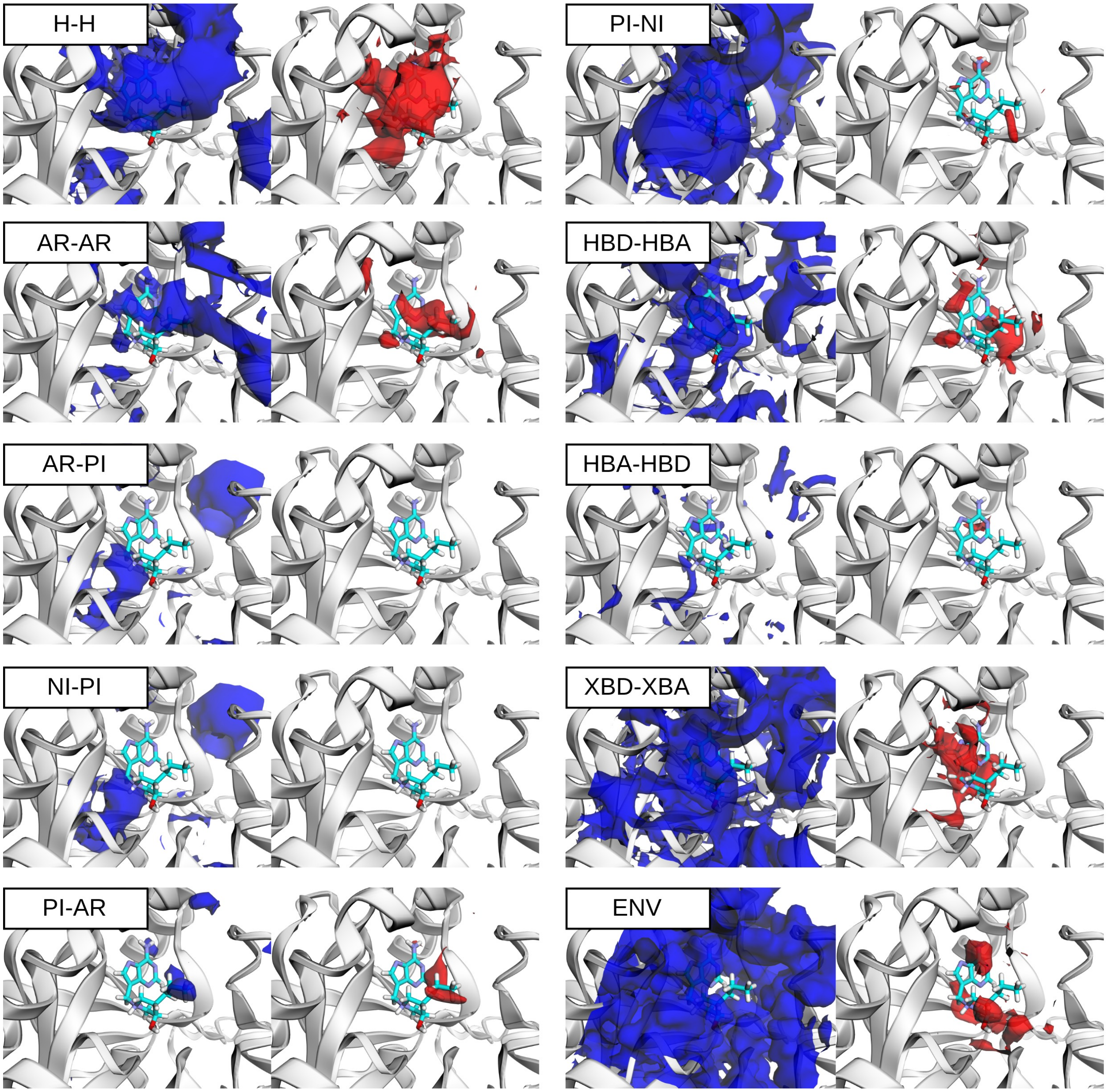

Figure S3: Visualization of GRAIL channels (blue, isosurface value 0.9) and corresponding attribution values (red, isosurface value 0.1) for the target 6AYO. Pharmacophoric features are categorized as hydrophobic (H), aromatic (AR), positive ionizable (PI), negative ionizable (NI), hydrogen bond donor (HBD), hydrogen bond acceptor (HBA), halogen bond donor (XBD), halogen bond acceptor (XBA). Channel notation follows the convention of [ligand-feature]-[binding-site-feature], for example, the HBA-HBD channel indicates that a ligand hydrogen bond acceptor is energetically favorable given the hydrogen bond donor moieties present in the binding site. ENV denotes atom density of the protein environment.

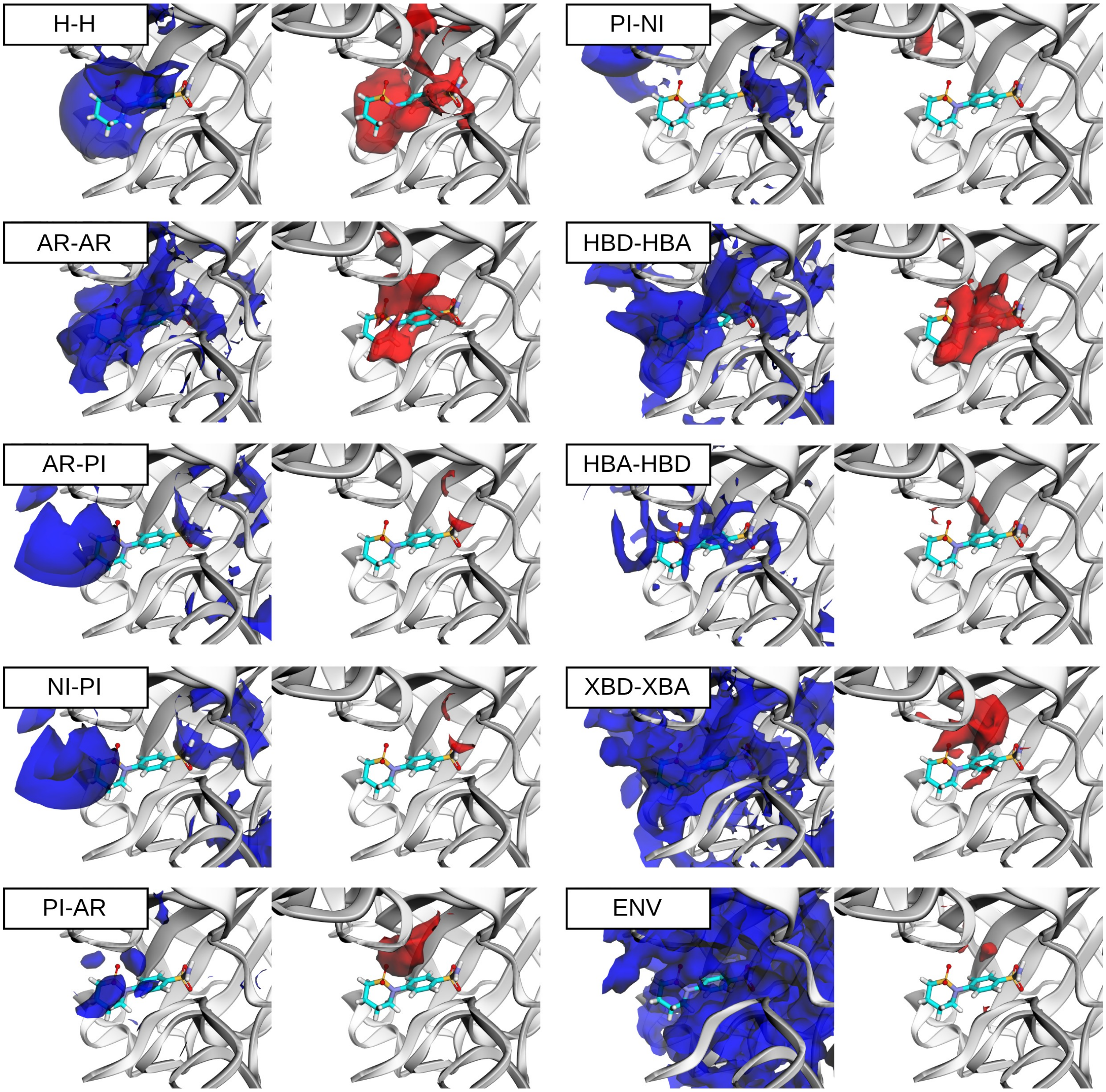

Figure S4: Visualization of GRAIL channels (blue, isosurface value 0.9) and corresponding attribution values (red, isosurface value 0.1) for the target 2Q1Q. Pharmacophoric features are categorized as hydrophobic (H), aromatic (AR), positive ionizable (PI), negative ionizable (NI), hydrogen bond donor (HBD), hydrogen bond acceptor (HBA), halogen bond donor (XBD), halogen bond acceptor (XBA). Channel notation follows the convention of [ligand-feature]-[binding-site-feature], for example, the HBA-HBD channel indicates that a ligand hydrogen bond acceptor is energetically favorable given the hydrogen bond donor moieties present in the binding site. ENV denotes atom density of the protein environment.

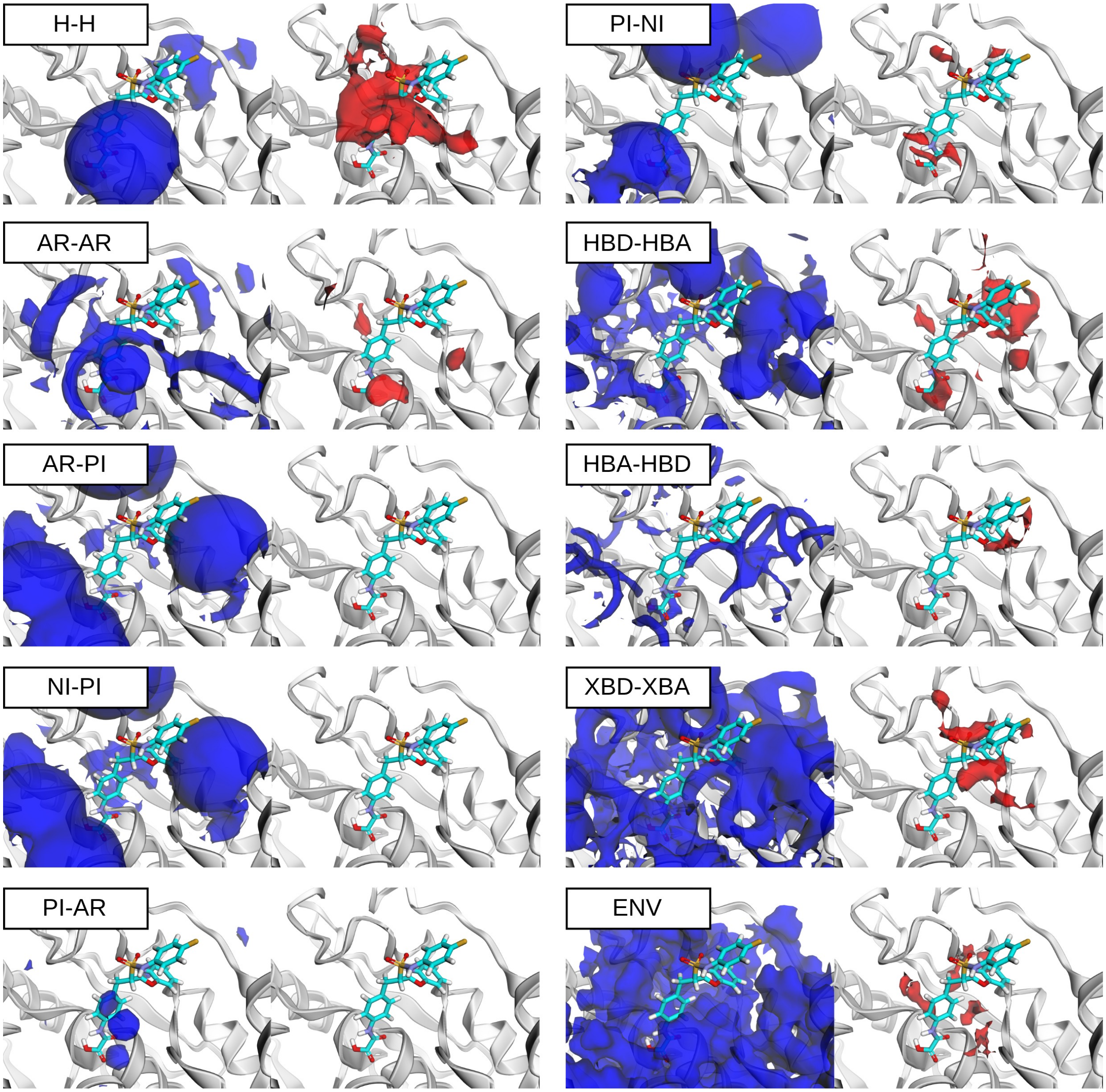

Figure S5: Visualization of GRAIL channels (blue, isosurface value 0.9) and corresponding attribution values (red, isosurface value 0.1) for the target 4I8N. Pharmacophoric features are categorized as hydrophobic (H), aromatic (AR), positive ionizable (PI), negative ionizable (NI), hydrogen bond donor (HBD), hydrogen bond acceptor (HBA), halogen bond donor (XBD), halogen bond acceptor (XBA). Channel notation follows the convention of [ligand-feature]-[binding-site-feature], for example, the HBA-HBD channel indicates that a ligand hydrogen bond acceptor is energetically favorable given the hydrogen bond donor moieties present in the binding site. ENV denotes atom density of the protein environment.

References

(1) Jiménez, J.; Skalic, M.; Martinez-Rosell, G.; De Fabritiis, G. K deep: protein–ligand absolute binding affinity prediction via 3d-convolutional neural networks. *J. Chem. Inf. Model.* **2018**, *58*, 287–296.

(2) Francoeur, P. G.; Masuda, T.; Sunseri, J.; Jia, A.; Iovanisci, R. B.; Snyder, I.; Koes, D. R. Three-dimensional convolutional neural networks and a cross-docked data set for structure-based drug design. *J. Chem. Inf. Model.* **2020**, *60*, 4200–4215.

(3) McNutt, A. T.; Francoeur, P.; Aggarwal, R.; Masuda, T.; Meli, R.; Ragoza, M.; Sunseri, J.; Koes, D. R. GNINA 1.0: molecular docking with deep learning. *J. Cheminform.* **2021**, *13*, 43.

(4) Ragoza, M.; Hochuli, J.; Idrobo, E.; Sunseri, J.; Koes, D. R. Protein–ligand scoring with convolutional neural networks. *J. Chem. Inf. Model.* **2017**, *57*, 942–957.

(5) Stepniewska-Dziubinska, M. M.; Zielenkiewicz, P.; Siedlecki, P. Development and evaluation of a deep learning model for protein–ligand binding affinity prediction. *Bioinformatics* **2018**, *34*, 3666–3674.

(6) Wang, Y.; Wei, Z.; Xi, L. Sfcnn: a novel scoring function based on 3D convolutional neural network for accurate and stable protein–ligand affinity prediction. *BMC bioinformatics* **2022**, *23*, 222.

(7) Jiménez, J.; Doerr, S.; Martínez-Rosell, G.; Rose, A. S.; De Fabritiis, G. DeepSite: protein-binding site predictor using 3D-convolutional neural networks. *Bioinformatics* **2017**, *33*, 3036–3042.

(8) Simonovsky, M.; Meyers, J. DeeplyTough: learning structural comparison of protein binding sites. *J. Chem. Inf. Model.* **2020**, *60*, 2356–2366.

(9) Storchi, L.; Cruciani, G.; Cross, S. DeepGRID: deep learning using GRID descriptors for BBB prediction. *J. Chem. Inf. Model.* **2023**, *63*, 5496–5512.

(10) Son, J.; Kim, D. Development of a graph convolutional neural network model for efficient prediction of protein-ligand binding affinities. *PloS one* **2021**, *16*, e0249404.

(11) Moon, S.; Zhung, W.; Yang, S.; Lim, J.; Kim, W. Y. PIGNet: a physics-informed deep learning model toward generalized drug–target interaction predictions. *Chem. Sci.* **2022**, *13*, 3661–3673.

(12) Zhang, X.; Gao, H.; Wang, H.; Chen, Z.; Zhang, Z.; Chen, X.; Li, Y.; Qi, Y.; Wang, R. Planet: a multi-objective graph neural network model for protein–ligand binding affinity prediction. *J. Chem. Inf. Model.* **2023**, *64*, 2205–2220.

(13) Satorras, V. G.; Hoogeboom, E.; Welling, M. E(n) equivariant graph neural networks. Proceedings of the 38th International Conference on Machine Learning. 2021; pp 9323–9332.

(14) Wang, K.; Zhou, R.; Tang, J.; Li, M. GraphscoreDTA: optimized graph neural network for protein–ligand binding affinity prediction. *Bioinformatics* **2023**, *39*, btad340.

(15) Kyro, G. W.; Smaldone, A. M.; Shee, Y.; Xu, C.; Batista, V. S. T-ALPHA: A Hierarchical Transformer-Based Deep Neural Network for Protein–Ligand Binding Affinity Prediction with Uncertainty-Aware Self-Learning for Protein-Specific Alignment. *J. Chem. Inf. Model.* **2025**, *65*, 2395–2415.

(16) Sverrisson, F.; Feydy, J.; Correia, B. E.; Bronstein, M. M. Fast end-to-end learning on protein surfaces. Proceedings of the IEEE/CVF conference on computer vision and pattern recognition (CVPR). 2021; pp 15272–15281.

(17) Li, Y.; Su, M.; Liu, Z.; Li, J.; Liu, J.; Han, L.; Wang, R. Assessing protein–ligand

interaction scoring functions with the CASF-2013 benchmark. *Nat. Protoc.* **2018**, *13*, 666–680.

(18) Su, M.; Yang, Q.; Du, Y.; Feng, G.; Liu, Z.; Li, Y.; Wang, R. Comparative assessment of scoring functions: the CASF-2016 update. *J. Chem. Inf. Model.* **2018**, *59*, 895–913.

(19) Wang, C.; Zhang, Y. Improving scoring-docking-screening powers of protein–ligand scoring functions using random forest. *J. Comput. Chem.* **2017**, *38*, 169–177.

(20) Trott, O.; Olson, A. J. AutoDock Vina: improving the speed and accuracy of docking with a new scoring function, efficient optimization, and multithreading. *J. Comput. Chem.* **2010**, *31*, 455–461.

(21) Li, J.; Guan, X.; Zhang, O.; Sun, K.; Wang, Y.; Bagni, D.; Head-Gordon, T. Leak Proof PDBBind: A Reorganized Data Set of Protein–Ligand Complexes for More Generalizable Binding Affinity Prediction. *J. Phys. Chem. B.* **2026**, *130*, 730–740, PMID: 41486605.

(22) Morgan, H. L. The generation of a unique machine description for chemical structures-a technique developed at chemical abstracts service. *J. Chem. Doc* **1965**, *5*, 107–113.

(23) Needleman, S. B.; Wunsch, C. D. A general method applicable to the search for similarities in the amino acid sequence of two proteins. *J. Mol. Bio.* **1970**, *48*, 443–453.

(24) Graber, D.; Stockinger, P.; Meyer, F.; Mishra, S.; Horn, C.; Buller, R. Resolving data bias improves generalization in binding affinity prediction. *Nat. Mach. Intell.* **2025**, *7*, 1713–1725.
